# Developmental Potential of embryos does not Impact Pregnancy Outcomes, but it Affects Live Birth Rates in Frozen Blastocyst Transfer Cycles

**DOI:** 10.5935/1518-0557.20210109

**Published:** 2022

**Authors:** Syed Waseem Andrabi, Puneet Rana Arora, Jaffar Mir, Satinder Kaur, Aarish Khan, Abdul Salam Albarki

**Affiliations:** 1 Milann-The Fertility Centre, New Delhi, India; 2 CIFAR- Centre for InFertility and Assisted Reproduction, Gurgaon, India; 3 Milann-The Fertility Centre, Chandigarh, India; 4 Sunrise Clinic Tripoli, Libya

**Keywords:** day-5 vs day- 6 blastocysts, frozen embryo transfer, clinical pregnancy rates, live-birth rates

## Abstract

**Objective:**

This study aimed to determine whether or not developmental potential impacts clinical outcomes, when good grade blastocysts from Days 5 and 6 were transferred in frozen embryo transfer (FET) cycles.

**Methods:**

654 women, including 460 (70.33%) on Day 5 and 194 (29.66%) on Day 6 were analyzed, in which 905 Day-5 and 274 Day-6 blastocysts were transferred. Only grade AA, AB, BA, BB quality and expansion grade between 3-6 (Gardner grading system) blastocysts survived and were included.

**Results:**

The implantation rate was higher, 41.9% (379/905) in normal Day-5 compared to delayed Day-6 blastocyst transfers - 36.5% (100/274), but not significant (*p*=0.1). The clinical pregnancy rate was similar and not significant (*p*=0.4) in normal Day-5 (32.4%), compared to delayed Day-6 (35%). Miscarriage rates were higher in normal Day-5 (13.3%) compared to delayed Day-6 (6.3%) blastocyst transfers but were not significant (*p*=0.06). On the other hand, the biochemical pregnancy rate was significantly higher (*p*=0.001) in the delayed Day-6 blastocysts (16.7%) transfer group compared to patients with normal Day-5 (2.4%) blastocyst transfers. Two patients had ectopic pregnancies from the delayed Day-6 blastocyst transfer group. Live-Birth rates were significantly higher in Day-5 blastocysts compared to Day-6 (*p*=0.03).

**Conclusions:**

The developmental potential of embryos should not be considered a negative influence on pregnancy outcomes, especially good grade blastocysts vitrified on Days 5 and 6. Fully expanded blastocysts on Day-5 are considered similar in terms of outcomes to delayed Day-6 blastocysts; however, live-birth rates are significantly higher in Day-5 blastocysts.

## INTRODUCTION

Almost forty-two years after the birth of the world's first in-vitro fertilization (IVF) baby, Louise Brown, more than eight million babies have been born globally as the result of assisted reproductive technologies (ART). After successful fertilization, the embryos can be cultured till Day 5 for blastocyst/s formation. Better culture conditions in the form of improved standard labs and media have an important role in blastocyst formation. Blastocyst stage culturing is not only the natural screening method, but it also helps us identify the best embryos with a higher potential of implantation. The first pregnancy report from blastocyst transfer was reported in 1985 (Cohen *et al*., 1985), and the first live birth in 1991 (Bolton *et al*., 1991). The trend of blastocyst transfers has increased in popularity since then and has been associated with higher implantation rates (Maheshwari *et al*., 2016). The higher implantation rates may be due to better synchronization between the endometrium and the transferred embryos or identifying the best embryos among the cohort at Day-5 or a combination of both. Blastocyst transfer not only results in increased implantation rates but also enables the transferring of fewer embryos compared to cleavage stage embryos; hence reducing multiple pregnancies, which in turn helps improving women health. Although, there are various complications associated with pregnancies of multiple gestations, including gestational diabetes, pre-eclampsia/hypertension, preterm birth, lower birthweight and continental malformations (Qin *et al*., 2015; 2016).

The development competence of an embryo is a multifactorial process. Embryos develop into the blastocyst stage on Day 5, however, some non-expanding embryos either in morula or cavitating morula stage are also found on Day 5 but when cultured to Day 6 for further expansion, it results in a blastocyst. Data suggest that synchrony between Day-6 blastocysts and endometrium may impair pregnancy rates in fresh transfer cycles, but several studies indicate a contrast in data obtained from Frozen Embryo Transfers (FETs). To improve the outcome in cases of delayed blastulation, extending the culture to Day 6 and freezing the embryos through vitrification provides the best chance for transferring a viable embryo as it achieves better endometrium-embryo synchronization. Apart from that, the live birth rates are higher with embryos transferred on the blastocyst stage, compared to the cleavage stage (Ozgur *et al*., 2015).

Vitrification, a well-established cryopreservation method, has proven to be very fruitful over the period. Recently, the trend of "freeze-all" and frozen embryo transfer have shown a gradual increase worldwide, suggesting an increase in pregnancy rates when frozen embryos are transferred compared to fresh transfers, especially in normal responders with oocyte numbers greater than > 10 (Boynukalin *et al*., 2020). In fresh cycles, we believe that stimulation impairs endometrial receptivity, perhaps one of the reasons for higher success rates of frozen-thawed embryo transfer (FET) (Shapiro *et al*., 2011).

## MATERIALS AND METHODS

This is a retrospective cohort analysis from September 2015 to December 2019 of couples undergoing ART treatment, with freeze-all blastocyst, followed by FBT cycles. We included 654 couples in this study, of which 460 were Day-5 and 194 were Day-6. We had 905 embryos transferred on the Day-5 group and 274 on the Day-6 group (Table 1). All the patients had primary infertility. Among 460 Day-5 patients, the main reason for infertility included 150 patients with tubal Factor (32.61%), 191 with Ovulation Dysfunction (including Diminished ovarian reserve) (41.52%), 32 had Endometriosis (6.96%), 15 had Uterine/Cervical factor (3.26%), and 72 had Unexplained infertility (15.65%). In the 194 Day-6 group of patients, 47 had tubal Factor (30%), Ovulation Dysfunction (including Diminished ovarian reserve) 98 (50.51%), Endometriosis 12 (6.18%), Uterine/Cervical factor 9 (4.64%), and Unexplained 28 (14.43%).

**Table 1.** Number of patients and embryos transferred in Day 5 and Day 6 groups.

TOTAL	Day 5	Day 6
Total number of patients	460	194
No. of embryos transferred	905	274

### Inclusion criteria

Couples with maternal age less than 40 years with prior stimulation, had their frozen embryos transferred both on days 5 and 6; had no expanded blastocyst on Day 5 (morulae or cavitating morulae), had at least one good fully expanded blastocyst at Day-6 for vitrification, thawing on Day-6, expanded blastocyst on Day-5 of HRT prepared endometrium, freeze-all cycles.

### Exclusion criteria

Fresh embryo transfer, slow freezing cycles with PGT-A, poor quality blastocysts, and male factor were excluded.

### Ovarian stimulation and Embryo Development

Two protocols, GnRH agonist protocol and GnRH antagonist short protocol were performed under pituitary suppression for ovarian stimulation. When at least a minimum of two follicles reaches the size of greater than 17mm in diameter, recombinant HCG 6500 IU was injected for the final maturation of oocytes. After 34-36 hours of induction of hCG, oocyte pickup was performed, and the oocytes were retrieved.

Prior to insemination, the oocytes were incubated for 2-4 hours. For insemination, we used both ICSI and conventional IVF techniques. After 15-18 hours of insemination, fertilization assessment was performed with two distinct pro-nuclei. In conventional IVF cases, the medium was changed to cleavage for further culture. Embryo quality was checked on Day 2 and subsequently on Day 3, and they were graded accordingly. Blastocyst culture was performed in cases with at least two, seven to nine cell grade A embryos present. On the morning of Day 5, we checked for blastocyst development. An afternoon assessment was done on blastocysts, which were not fully expanded. In cases where no expanded blast was present till Day 5 evening, we extended the culture to Day 6. On Day 6, after an early morning and followed by afternoon blastocyst assessment, all good grade expanded blastocysts were vitrified. All the cycles included freeze-all.

### Blastocyst Grading

We graded the morulae as top quality, good quality, average quality and poor quality (Tao *et al.*, 2012). Embryos in which all blastomeres were compact and resulted in the shape of a smooth sphere were termed as top-quality morula. In good quality, more than 75% of blastomere underwent compaction with a shallow indent. However, in average quality morulae, up to 75% blastomeres compact, there was a deep indentation. In poor quality morulae, 30% of blastomeres showed compaction, forming one, two or more lobes (Tao *et al*., 2012).

As per the Gardner criteria, depending upon the expansion of blastocyst, development of inner cell mass (ICM) and appearance of trophectoderm (TE), blastocyst grading was performed. Six grades were given to the blastocysts, depending upon the degree of expansion: (1) non-expandable, less than 50% blastocoel filling; (2) more than 50% blastocele filling; (3) entire blastocyst filled with blastocoel but not expanded, and with a thick zona-pellucida; (4) fully expanded blastocyst with a thin zona; (5) fully expanded hatching blastocyst; (6) a complete hatched blastocyst. ICM was graded as A, B and C. Blastocysts with A grade ICM had tightly packed cells in mushroom-style; in B grade ICM, the cells were loosely gathered, while in the C grade no cells were identifiable. TEs were also graded as A, B and C. In A grade, an epithelial cohesive layer was established by many cells. In the B grade, the loose epithelium was created by few cells and C grade TE had few large epithelial cells (Gardner *et al*., 2004). Depending upon the expansion stage, 1 and 2 were very hard to grade as no ICM and TE can be distinguished. Once the blastocoel cavity starts developing, we assessed the ICM and TE. Depending upon ICM and TE morphology, the blastocysts were grouped into the four main categories: excellent (3AA, 4AA, 5AA, and 6AA), good (3AB, 4AB, 5AB, 6AB, 3BA, 4BA, 5BA, and 6BA), average (3BB, 4BB, 5BB, and 6BB), and poor (3BC, 4BC, 5BC,6BC, 3CB, 4CB, 5CB, 6CB, 3CC, 4CC, 5CC, and 6CC). All the blastocysts were scored before vitrification and before transfer after thawing.

### Vitrification and warming procedures

Expanded blastocysts were suspended in equilibration solution for 12 to 15 minutes at room temperature and then transferred to vitrification solution for 50 seconds and then loaded on vitrification devices which were labelled properly with identification numbers and name of the couple. The volume of vitrification solution used for loading was minimal (just to cover the blastocysts). Once loaded, the device was plunged immediately into liquid nitrogen directly and finally stored in cryogenic storage vessels. Not more than two blastocysts were vitrified in one cryo-device.

For warming, at 37°C, the blastocysts were placed in a thawing solution for one minute, followed by blastocyst transfer to a diluted solution for 3 minutes, then to a washing solution for 5 minutes and finally in a transfer media for at least 2 hours of incubation for re-expansion and survival check.

### Endometrial preparation for vitrified warming cycles

We started endometrial priming on Day2/Day3 of the menstrual cycle with oral estradiol valerate, starting with 2mg three times a day. We ran an ultrasound scan a week later and if the endometrial thickness was found to be less than 6mm, the dose was increased to 4 mg twice a day orally and 4mg vaginally (afternoon dose). There was estradiol exposure for 9-14 Days. Once endometrial thickness reached 7.5mm and more, a vaginal micronized preparation of progesterone was started with 400 mg twice-a-day. Embryo transfer was planned after five Days of exposure to progesterone. In cases where endometrial thickness did not reach 7 mm even after 25 days of exposure to estradiol valerate, the embryo transfer cycle was cancelled to be rescheduled for endometrial preparation again with a different protocol.

### Clinical outcomes

We checked hCG Serum levels after 14 days of embryo transfer. Levels above 100 IU/l were considered positive, and those below 100 as biochemical, in case it didn't double in 48-72 hours. Gestational sac after 6 weeks by sonography was considered as a clinical pregnancy. Live birth rate was considered as any birth happening after 24 weeks of pregnancy. All multiple pregnancy deliveries were counted as one live birth.

### Embryo Transfer

Patients with at least one expanded blastocyst with distinct ICM and well-defined blastocoel cavity filling the embryo were considered for transfer. All Day-6 vitrified blastocysts were thawed at Day-5 of progesterone. All the thawed Day-5 or Day-6 blastocysts were incubated for at least an hour before transfer to check the viability.

### Statistical Analysis

Statistical analysis was done in SPSS version 11. Patient age, number of oocytes retrieved, the percentage of oocytes fertilized, number of embryos developing to the blastocyst stage, the number of embryos transferred, and all clinical parameters were compared with the t-test. *P* ≤ 0.05 was considered statistically significant.

## RESULTS

A total of 654 FET cycles including 460 Day-5, and 194 Day-6 met our inclusion criteria. The analysis was carried out on blastocysts that successfully survived after thawing. There were no notable statistical differences between Day-5 and Day-6 transfers with respect to age, number of oocytes retrieved, mature oocytes and fertilization rates (*p*>0.05) (Table 2). However, the number of blastocysts vitrified on either Day 5 or Day 6 were found to be significantly higher in Day 5 compared to Day 6 (*p*=0.001). Similarly, the number of embryos transferred was found to be significant (*p*=0.001) between the two groups, since more embryos were transferred in mixed Day 5 compared to the Day 6 (Table 3).

**Table 2.** Comparison of different parameters between Day 5 and Day 6  FETs .

Sl.No.		Days	Mean	S.D	SE	*p* value
1	Age	Day 5	33.1	5.2	0.2	0.4
		Day 6	33.4	4.6	0.3	
2	No. of oocytes retrieved	Day 5	13.8	7.7	0.4	0.1
		Day 6	13.5	5.9	0.4	
3	MII	Day 5	10.8	6.2	0.3	0.07
		Day 6	10.5	4.7	0.3	
4	Fertilization Rates	Day 5	8.7	5.3	0.2	0.4
		Day 6	8.5	4.3	0.3	
5	Embryos frozen	Day 5	4.4	2.8	0.1	0.001
		Day 6	3.2	1.6	0.11	

**Table 3.** Overall clinical outcomes embryo transfer done on Day 5 and Day 6 embryo transfer.

TOTAL	Day 5 (n=460)	Day 6 (n=194)	*p* value
No. of Embryos transferred	905	274	
No. of Embryos implanted [n (%)]	378 (41.76%)	101 (36.86%)	0.1
Positive [n (%)]	293 (63.70%)	96 (49.48%)	0.4
Biochemical [n (%)]	7 (2.4%)	16 (16.67%)	**0.001**
Miscarriages [n (%)]	39 (13.31%)	6 (6.25%)	0.06
Ectopic [n (%)]	0 (0)	2 (2.08%)	**0.01**
Live Birth rates [n (%)]	247 (53.70%)	72 (37.11%)	**0.03**

Upon comparing the clinical parameters among the two groups, the implantation rates were found similar in Day 5 (41.9%) and Day 6 (36.5%) (*p*=0.1). The positive pregnancies were higher in Day 5 blastocyst transfers, constituting 293 out of 460 (63.70%) compared to Day 6, 96 out of 194 (49.48%); however, non-significant (*p*=0.4). There were no significant differences in overall miscarriage rates between Days 5 (13.31%) and 6 (6.25%) frozen blastocyst transfers. There was a significant difference in biochemical pregnancies (*p*=0.001) between the two groups, as Day 6 blastocysts transfers (16.67%) have significantly higher (*p*=0.001) biochemical pregnancies compared to Day 5 blastocyst transfers (2.4%). There were no ectopic pregnancies in Day 5 blastocyst transfers; however, 2 ectopic pregnancies were found in Day 6. A significant difference was seen in overall live birth rates between the two groups (*p*=0.03), as Day 5 had higher birth rates compared to Day 6.

Upon further analyzing 460 Day 5 blastocyst transfers - 66 (14.35%) couples underwent SET; in which 34 (51.51%) showed successful implantation; while 394 (85.65%) had two blastocysts transfers with 241 (61.17%) successful implantations. Similarly, in the Day-6 group, 67 (34.54%) couples had SET, while 127 (65.46%) opted for two embryo transfers (Table 4).

**Table 4.** Single embryo transfers in Day 5 and Day 6.

	Total No. of Transfers	Positive
**Day 5** **SET** **2-ET** **Total**	66 394 460	34 241 275
**Day 6** **SET** **2-ET** **Total**	67 127 194	29 69 98

## DISCUSSION

The primary goal of IVF treatment is a healthy viable singleton delivery. Recently, this goal has been achieved by transferring embryo/s that are morphologically best grade or by performing genetic screening. The recent increased trend of freeze-all has overall improved outcomes could be as a result of better vitrification-thawing media, improvements in culture condition, better embryo selection, and embryo-endometrium synchronization. The recent trend of single embryo transfer (SET) is on the rise; however, many centers still opt for multiple embryo transfers, especially with advanced maternal age (Gleicher & Barad, 2009). In embryo cohorts of patients undergoing ART treatment, normal development of blastocysts at Day-5 is sometimes slowed/delayed and can result in fresh transfer cancellation. In such slow development cases, the embryo is cultured for day-6 for freezing and transferred on day 5 to the endometrium in the FET cycle.

Frozen embryo transfer was included in our study to avoid the developmental stage difference between embryos and endometrium, and ovarian hyper stimulation syndrome. Our study evaluated the different clinical outcomes after transferring blastocysts on days 5 or 6. There was a significant increase in the implantation rate on day-5 transfer (59.8%) as compared to day-6 transfer (50.5%) (*p*=0.02). Similarly, Khorram *et al*. (2000), Shoukir *et al*. (1998), Shapiro *et al*. (2001), and Hashimoto et al. (2013) have also shown that embryos expanded on day 5 have a two-fold increased implantation rate than day-6 embryos. Further, Desai *et al*. (2016) reported a three-fold increase in clinical pregnancy rates after the transfer of day-5 blastocysts, as compared to day-6 blastocysts. Hashimoto *et al.* (2013) also found increased pregnancy rates in day-5 embryos transfer. The reason for implantation failure in slow-growing embryos (i.e., day 6) may be due to a higher incidence of abnormal spindles (Haas *et al*., 2016). However, in the meta-analysis involving 15 studies (Sunkara *et al*., 2010) reported significantly higher CPRs and ongoing pregnancy rates for Day-5 compared to Day-6 transfers in cryopreserved blastocysts. Subsequently, both slow-freezing and vitrification cycles showed that implantation, clinical and ongoing pregnancy rates were significantly increased for day-5 versus day-6 transfers (Kovalevsky *et al*., 2013).

Elgindy & Elsedeek (2012) showed that early expanded blastocysts, when transferred on Day 5 or Day 6, blastocysts had a similar pregnancy rate; but there was a significantly lower pregnancy rate in the late expanded blastocyst group (i.e., Day-6 blastocyst) and transferred on Day-6. Hashimoto and his colleges demonstrated a similar result, where slow-growing embryos showed a lower pregnancy rate when compared to the normally developing embryos. They also concluded that the incidence of abnormal spindles in the slow-growing embryos was significantly higher than the normally developing embryos (Hashimoto *et al*., 2013). A meta-analysis concluded that the transfer of Day-5 and Day-6 embryos with similar morphological quality showed a significant increase in the pregnancy rate on Day-5 as compared to Day-6 (Sunkara *et al*., 2010). We demonstrated that the pregnancy rate is significantly lower during FET cycles with Day-6 vitrified blastocysts, even if they were morphologically graded as good-quality embryos compared to blastocysts vitrified on Day-5, which was like the previous studies. Moreover, the vitrified blastocysts on Day-6 were of higher quality compared to the blastocyst vitrified on Day-5 but still resulted in a significantly lower pregnancy rate.

The studies have shown that bioenergetic issues and higher aneuploidy rates were common in slow-growing embryos (Capalbo *et al*., 2014; Yin *et al*., 2015). The intrinsic weakness of slow-growing embryos remains unclear. It may be due to variations in laboratory procedures, endometrial preparation, culture medium, vitrification, and warming procedures. Taylor *et al*. calculated that blastocysts that formed and were vitrified on Day-6 (slow-developing embryos) had a 10% increase in aneuploidy rates compared with those that formed on Day 5 (Taylor *et al*., 2014). Besides, studies have also shown that euploidy rates were similar in the Day-5 and Day-6 embryo groups (Alfarawati *et al*., 2011; Fragouli *et al*., 2014). But others showed that delayed blastulation is not associated with increased aneuploidy rates, and postulated absence of blastulation is associated with increased aneuploidy (Kroener *et al*., 2012). On the contrary, Capalbo *et al*. (2014) demonstrated that faster-growing embryos (Day-5 blastocysts) showed higher euploidy rate as compared with slower-growing ones (Day-6 blastocysts). Further, the euploid rates of Day-5 blastocysts were higher than Day-6 blastocysts and the outcomes were similar after transferring euploid Day-5 or euploid Day-6 blastocysts (Taylor *et al*., 2014). In the present study we did not test for blastocyst euploidy; therefore, we could not investigate whether the differences between the Day-5 and Day-6 groups were related to a different rate of chromosomal abnormalities.

The present study has the limitation of its retrospective design and could not contribute to understanding the problems of slow-delayed growth embryos/blastocysts. However, we added evidence of the clinical advantage of Day-5 and Day-6 vitrification and single-embryo transfer setting. We observed that Day-6 blastocyst transfers resulted in a significant increase in biochemical pregnancies (16.33%), as compared to Day-5 (2.55%). But there were no differences concerning clinical and ectopic pregnancy rates between Day-5 and Day-6 vitrified and frozen embryos. Further, the Day-5 embryo transfers showed an increased miscarriage rate (14.2%), as compared to Day-6 (6.12%). However, we also observed that Day-6 blastocyst transfers have resulted in clinical pregnancies. Therefore Day-6 blastocyst transfer remains a viable option if good quality Day-6 blastocysts are available. Future studies must be done to know the live-birth rates and long-term postnatal outcomes after the transfer of slowly developed and late-vitrified blastocysts.

## CONCLUSION

In conclusion, the main limitation of the present study is a disproportionate sampling and multiple blastocyst transfer. Even though, clinical outcomes in Day-5 blastocyst transfers are higher compared to Day-6. This study cannot be generalized to say that Day-5 blastocyst transfer is better than that of Day-6, due to the limitation of not transferring a single blastocyst, which should be ideal to give us the exact outcome details. Also, at the time of embryo transfer, couples are counselled and their strong desire of getting conceived by IVF goes in favor of transferring more than one embryo. The present study can be an add-on to the past studies - which proves that live-birth rates are better in Day-5 embryo transfers compared to Day-6.
